# Biochemical characterization of the first step in sulfonolipid biosynthesis in *Alistipes finegoldii*

**DOI:** 10.1016/j.jbc.2022.102195

**Published:** 2022-06-25

**Authors:** Christopher D. Radka, Darcie J. Miller, Matthew W. Frank, Charles O. Rock

**Affiliations:** 1 Department of Infectious Diseases, St. Jude Children’s Research Hospital, Memphis, Tennessee, USA; 2 Department of Structural Biology, St. Jude Children’s Research Hospital, Memphis, Tennessee, USA

**Keywords:** sulfonolipid biosynthesis, condensing enzyme, 3-ketocapnine, *Alistipes finegoldii*, fatty acid, crystal structure, Bacteroidetes, gut microbiome, ACP, acyl carrier protein, AOS, α-oxoamine synthase, PLP, pyridoxal phosphate, Spt, serine palmitoyltransferase, SulA, cysteate acyl-ACP transferase

## Abstract

Sulfonolipids are unusual lipids found in the outer membranes of Gram-negative bacteria in the phylum Bacteroidetes. Sulfonolipid and its deacylated derivative, capnine, are sulfur analogs of ceramide-1-phosphate and sphingosine-1-phosphate, respectively; thus, sulfonolipid biosynthesis is postulated to be similar to the sphingolipid biosynthetic pathway. Here, we identify the first enzyme in sulfonolipid synthesis in *Alistipes finegoldii* as the product of the *alfi_1224* gene, cysteate acyl-acyl carrier protein (ACP) transferase (SulA). We show SulA catalyzes the condensation of acyl-ACP and cysteate (3-sulfo-alanine) to form 3-ketocapnine. Acyl-CoA is a poor substrate. We show SulA has a bound pyridoxal phosphate (PLP) cofactor that undergoes a spectral redshift in the presence of cysteate, consistent with the transition of the lysine–aldimine complex to a substrate–aldimine complex. Furthermore, the SulA crystal structure shows the same prototypical fold found in bacterial serine palmitoyltransferases (Spts), enveloping the PLP cofactor bound to Lys251. We observed the SulA and Spt active sites are identical except for Lys281 in SulA, which is an alanine in Spt. Additionally, SulA(K281A) is catalytically inactive but binds cysteate and forms the external aldimine normally, highlighting the structural role of the Lys281 side chain in walling off the active site from bulk solvent. Finally, the electropositive groove on the protein surface adjacent to the active site entrance provides a landing pad for the electronegative acyl-ACP surface. Taken together, these data identify the substrates, products, and mechanism of SulA, the PLP-dependent condensing enzyme that catalyzes the first step in sulfonolipid synthesis in a gut commensal bacterium.

Sulfonolipids are an unusual class of bacterial lipids that are structurally related to phosphorylated sphingolipids (Fig. 1*A*). Sulfobactin A arises from N-acylation with a 3-hydroxy-acyl-acyl carrier protein (ACP) and sulfobactin B arises from acyl-ACP acylation. Sulfonolipids were first identified in 1975 in the lipids of *Nitzschia alba*, a nonphotosynthetic marine diatom (algae) (1), and are found in several environmental Bacteroidetes genera (2, 3, 4, 5, 6), Bacteroidetes associated with the oral microbiome (7), and Bacteroidetes associated with the gut microbiome (8, 9). Cellular fractionation experiments with the environmental Bacteroidetes *Cytophaga johnsonae* localize sulfonolipids to the outer membrane (2, 6, 10, 11, 12, 13). The deacylated form of sulfonolipids (capnine) is structurally related to sphingosine-1-phosphate (Fig. 1*B*) and is found in the lipids of *Capnocytophaga* species from the oral microbiome. The variation in capnine abundance among different strains of *Capnocytophaga* suggest capnine is not just a transient intermediate of sulfonolipid synthesis in these bacteria (7). A metabolomic analysis of mouse intestine detected sulfonolipids and capnine in cecal extracts, and *Alistipes* and *Odoribacter* species are the most prominent members of the gut commensal Bacteroidetes community that produce sulfonolipids (9). It is not clear if cecal capnine is a bacterial membrane component or arises from the digestion of sulfonolipid. Sulfonolipids support gliding motility in environmental Bacteroidetes (14, 15) and trigger multicellular development in choanoflagellates (16, 17), the closest living relatives of animals. Sulfonolipids suppress inflammation in mouse models (18), suggesting a role for sulfonolipids produced by gut commensals in regulating the immune response. The anti-inflammatory properties of sulfonolipids may explain why fecal transplantation with commensal sulfonolipid-producing *Alistipes* species prolonged skin graft survival in mice (19). *Alistipes* and *Odoribacter* species, but not other sulfonolipid-free Bacteroidetes species, prevent inflammatory bowel disease (20, 21) suggesting a connection to sphingosine-phosphate receptor signaling, which is integral to the development of gut inflammation (22).

**Figure 1:**
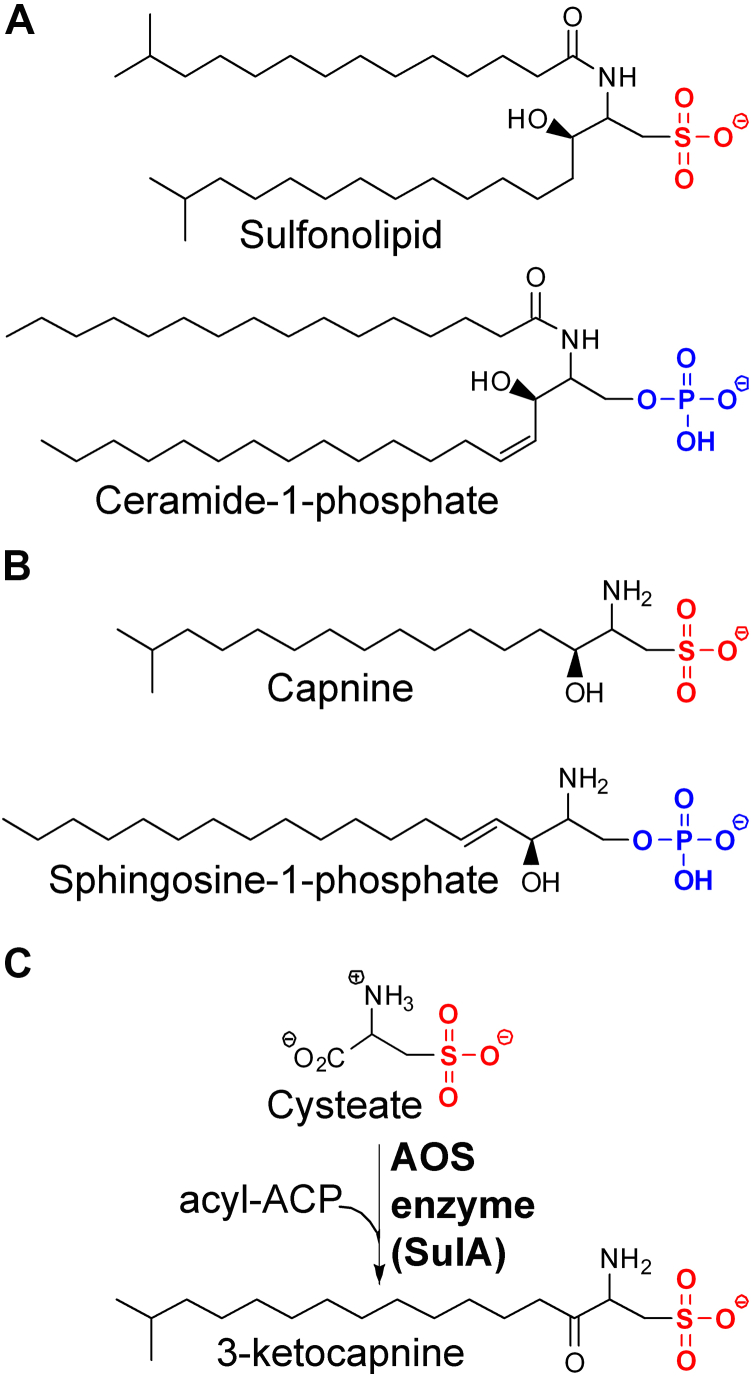
**Sulfonolipids are structurally related to sphingolipids.***A*, sulfonolipid (sulfobactin B) is structurally related to ceramide-1-phosphate. Sulfobactin A has an amide-linked 3-hydroxy-acyl chain. *B*, capnine is a nonhydrolyzable analog of sphingosine-1-phosphate. *C*, the sulfonolipid biosynthetic pathway is analogous to the bacterial sphingolipid biosynthetic pathway (28). The first step is carried out by a condensing enzyme belonging to the α-oxoamine synthase (AOS) protein superfamily to form 3-ketocapnine (2-amino-3-keto-15-methylhexadecane-1-sulfonic acid) (SulA). There are two genes encoding members of this protein family in *A. finegoldii* (*alfi_1224* and *alfi_1465*). SulA, cysteate acyl-ACP transferase.

The structural similarities between sulfonolipid and sphingolipids led White (23) to propose a sulfonolipid biosynthetic pathway beginning with the condensation of cysteate (7, 23, 24, 25, 26) with acyl-CoA to form 3-ketocapnine. A survival screen identified the structural genes for a condensation enzyme (Spt), a ketoreductase (CerR), and an N-acyltransferase (CerS) that are required for ceramide synthesis in *Caulobacter* (27). Stankeviciute *et al.* (28) identified the biochemical function of each gene *in vitro* and validated the conclusions with measurements of the accumulated cellular intermediates in specific pathway knockout strains of *Caulobacter crescentus*. These data show the sequence of reactions in the bacterial sphingolipid biosynthetic pathway is not the same as in mammals in that the ketoreductase step occurs after the acyltransferase step rather than before the acyltransferase as in the human pathway. The formation of 3-ketocapnine by Spt-related proteins derived from *Capnocytophaga ochracea* (29) and *Chryseobacterium gleum* (30) is dependent on acyl-CoA and cysteate. Liu *et al.* (29) suggest that the bacterial sulfonolipid pathway is different from the bacterial sphingolipid pathway because there is a ketoreductase that produces capnine after the condensation step, rather than after the acylation step. However, the ketoreductase employed in the biochemical assay was not genetically validated as a component of the sulfonolipid biosynthetic pathway. Vences-Guzman *et al.* (31) recently reported that deletion of a Spt-related gene (Fjoh_2419) in the environmental Bacteroidetes *Flavobacterium johnsoniae* eliminated sulfonolipid production. They propose the enzyme is responsible for the formation of capnine, not 3-ketocapnine, although the product of the reaction was not structurally characterized, and the assay system used did not contain cysteate. Lee *et al.* (32) identified an enzyme (BT_0972) with 3-ketosphinganine reductase activity from a biochemical screen, and the amino acid sequence contains conserved features of NAD(P)H-dependent short-chain dehydrogenases/reductases. Additional experiments are needed to validate the sequence of reactions in bacterial sphingolipid synthesis; however, regardless of the sequence of steps, the consensus view is that the first step in sulfonolipid synthesis is catalyzed by an Spt-like condensation enzyme that remains to be biochemically or structurally characterized (29, 30, 31) (Fig. 1*C*). In this prior research, acyl-CoA was used as the donor in the condensation reaction (27, 29, 30, 31); however, it seems most likely that acyl-CoA is a substrate analog and that the actual acyl donors for sphingolipid and sulfonolipid synthesis are attached to ACP, the biosynthetic acyl group carrier in bacteria.

The goal of this study is to biochemically and structurally characterize the first step in *Alistipes finegoldii* sulfonolipid biosynthesis. *A. finegoldii* is an anaerobic human gut commensal bacteria and the gene that encodes the cysteate acyl-ACP transferase (SulA) was identified using a biochemical screen. Purified SulA catalyzed the formation of 3-ketocapnine using acyl-ACP and cysteate as substrates. SulA is specific for cysteate, which forms an external aldimine with the bound pyridoxal-5′-phosphate (PLP) cofactor. Acyl-ACP is the physiological acyl donor, whereas acyl-CoA is a low-affinity substrate analog. The SulA X-ray crystal structure shows it adopts the same fold as bacterial Spts but differs from these enzymes in having Lys281 in the active site instead of alanine. SulA(K281A) is catalytically inactive but forms the external cysteate aldimine normally highlighting the structural role for the Lys281 in forming the active site. SulA also has a pronounced positively charged surface feature adjacent to the active site entrance that complements the electronegative ACP surface. This work identifies the structure, substrates, and product of the first step in sulfonolipid synthesis in a gut commensal bacterium and identifies Lys281 as a key residue that distinguishes SulA from Spt.

## Results

### Identification of SulA

Our bioinformatic analysis (8) revealed two genes (*alfi_1224* and *alfi_1465*) in *A. finegoldii* encoding enzymes belonging to the α-oxoamine synthase (AOS) protein superfamily of PLP-dependent enzymes (33). The α-oxoamine synthase protein family catalyze the stereospecific carbon-carbon bond formation between the α-carbon of an α-amino acid and the carbonyl carbon of a thioester. The *alfi_1224* gene encodes a protein in the Spt subgroup of AOS enzymes and *alfi_1465* encodes a protein most closely related to the 2-amino-3-ketobutyrate coenzyme A ligase subgroup (Fig. S1). The *fjoh_2419* gene from *F. johnsoniae* is a close relative of *aIfi*_1224 in the Spt arm of the AOS enzyme family (Fig. S1). Bacterial Spt enzymes have been measured using acyl-CoA as substrate (34, 35, 36, 37). Acyl-CoA is the correct substrate for eukaryotic Spt; however, it seems likely that acyl-CoAs are acting as substrate analogs in the bacterial Spt enzymes because the biologically relevant acyl group carrier in bacterial lipid synthesis is ACP. Acyl-ACP is the established acyl donor for membrane lipid synthesis in *A. finegoldii* (8), therefore, we prepared [^14^C]16:0-ACP (Fig. S2) as the substrate to detect SulA activity.

**Figure 2:**
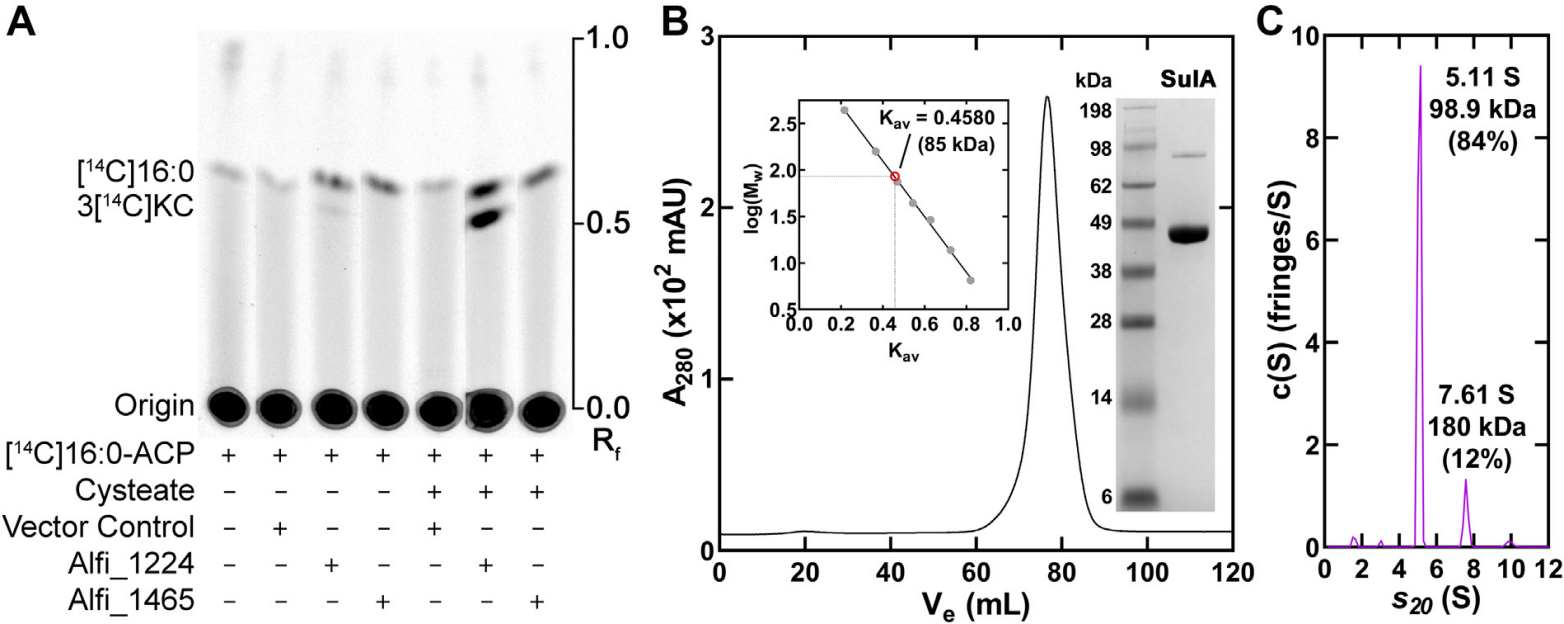
**Identification and purification of cysteate acyl-ACP transferase.***A*, TLC separation of 3-keto[^14^C]capnine (3[^14^C]KC) arising from SulA activity using cellular lysates from *Escherichia coli* cells expressing nothing (empty vector control), *alfi_1224* (SulA), or *alfi_1465*. The thin-layer plate is representative of four independent experiments. *B*, purified SulA migrates as a single 85 kDa species (*V*_*e*_ = 76.6 ml) based on a calibrated HiLoad 16/600 Superdex 200 column (98.1 kDa theoretical homodimer mass). *Left inset*, molecular weight calculation using calibration standards described under Experimental procedures. *Right inset*, SDS gel electrophoresis shows SulA purity (∼48 kDa) along with molecular weight standards. *C*, the sedimentation velocity profile (fringe displacement) was fit to a continuous sedimentation coefficient distribution model c(S). SulA sedimented as a 98.9 kDa homodimer with a sedimentation coefficient of 5.11 S. ACP, acyl carrier protein; SulA, cysteate acyl-ACP transferase.

The *alfi_1224* and *alfi_1465* genes were expressed in *Escherichia coli* and cell lysates were prepared. These lysates were incubated with [^14^C]16:0-ACP either with or without cysteate and the products separated by TLC (Fig. 2*A*). The [^14^C]16:0-ACP substrate is at the origin of the thin-layer plate and there is a detectable amount of [^14^C]16:0 released from [^14^C]16:0-ACP by hydrolases in all of the *E. coli* cell lysates. There is a new radiolabeled lipid detected in lysates from cells expressing *alfi_1224* that is dependent on the presence of cysteate in the assay (Fig. 2*A*). Based on these biochemical characteristics, this product was tentatively identified as 3-keto[^14^C]capnine and the enzyme named SulA.

His-tagged SulA was purified by nickel affinity chromatography and gel filtration chromatography to >95% purity based on gel electrophoresis (Fig. 2*B*). The elution position in the gel filtration experiment indicated it is a homodimer, like bacterial Spt (35). Sedimentation velocity experiments with SulA confirmed its dimeric state, although a percentage of SulA did exist as a tetramer (Fig. 2*C*). The frictional ratio of *f*/*f*_*0*_ = 1.35 is consistent with SulA being a globular protein (38).

### SulA binds PLP

**Figure 3:**
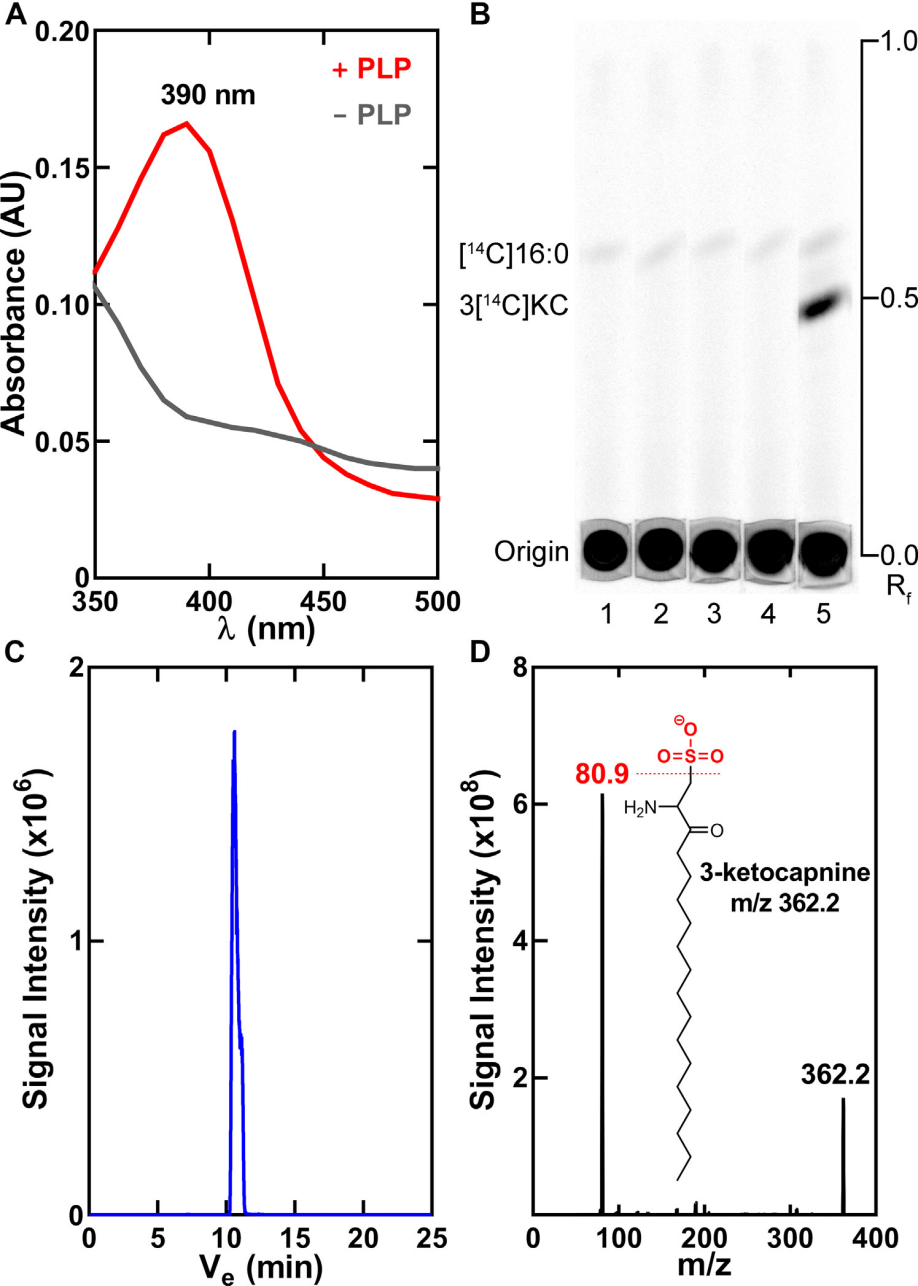
**SulA activation and product identification.***A*, absorption spectra of SulA before (−PLP) and after (+PLP) loading by dialysis. Based on the extinction coefficient of the PLP absorbance at 390 nm, >95% of SulA is in the internal aldimine state following PLP loading. *B*, SulA is specific for cysteate. Lane 1, [^14^C]16:0-ACP; lane 2, [^14^C]16:0-ACP + SulA; lane 3, [^14^C]16:0-ACP + SulA + serine; lane 4, [^14^C]16:0-ACP + SulA + phosphoserine; and lane 5, [^14^C]16:0-ACP + SulA + cysteate. Assays and TLC were carried out as described under Experimental procedures. The thin-layer plate is representative of three independent experiments. *C*, LC-MS analysis of a scaled up SulA reaction revealed a new acyl-ACP– and cysteate-dependent peak in the total ion current at the 10.4 min region of the gradient. *D*, analysis of the reaction product by mass spectrometry confirms the formation of 3-ketocapnine (*m/z* = 362.2) with the diagnostic fragmentation pattern showing the loss of the sulfonic acid (HSO_3_) headgroup (*m/z* = 80.9). ACP, acyl carrier protein; PLP, pyridoxal phosphate; SulA, cysteate acyl-ACP transferase.

Amino acid sequence comparison of SulA with other AOS protein family members showed the strict conservation of key PLP-binding residues (Fig. S3), indicating that SulA is a PLP-dependent enzyme. SulA protein preparations have a slight yellow coloration that intensified during protein concentration suggesting SulA copurifies with some PLP obtained from the *E. coli* expression system. However, the UV-Vis spectrum of the protein sample showed a weak PLP absorbance (Fig. 3*A*). Bacterial Spt are efficiently loaded with PLP by dialysis (34, 35), and we successfully employed this technique to load PLP onto SulA (Fig. 3*A*). The PLP absorbance maxima at 390 nm represents the internal aldimine with a SulA lysine and we calculate that >95% of SulA is in the PLP internal aldimine state following PLP loading. This active enzyme preparation was used in the biochemical experiments. Purified SulA produces the same labeled product, 3-keto[^14^C]capnine, as observed in the cell lysate experiments. Serine and phosphoserine are not substrates for SulA, whereas cysteate robustly supports the reaction (Fig. 3*B*).

### SulA forms 3-ketocapnine

LC-MS was used for analysis of SulA reactions with 50 μM 16:0-ACP to positively identify the reaction product. A new ion peak appeared at 10 min in the gradient in SulA reactions containing acyl-ACP and cysteate that is absent from reactions without one or both substrates (Fig. 3*C*). The product had a parent mass of *m/z* = 362.2 consistent with the condensation of a 16-carbon acyl chain with cysteate coupled with the loss of CO_2_ from the cysteate carboxyl (Fig. 3*D*). The major fragment has an *m/z* = 80.9, which is diagnostic for the loss of the sulfonic acid (HSO_3_) headgroup (Fig. 3*D*) (8). These results positively identify 3-ketocapnine as the SulA product.

### Cysteate and acyl-ACP are SulA substrates

**Figure 4:**
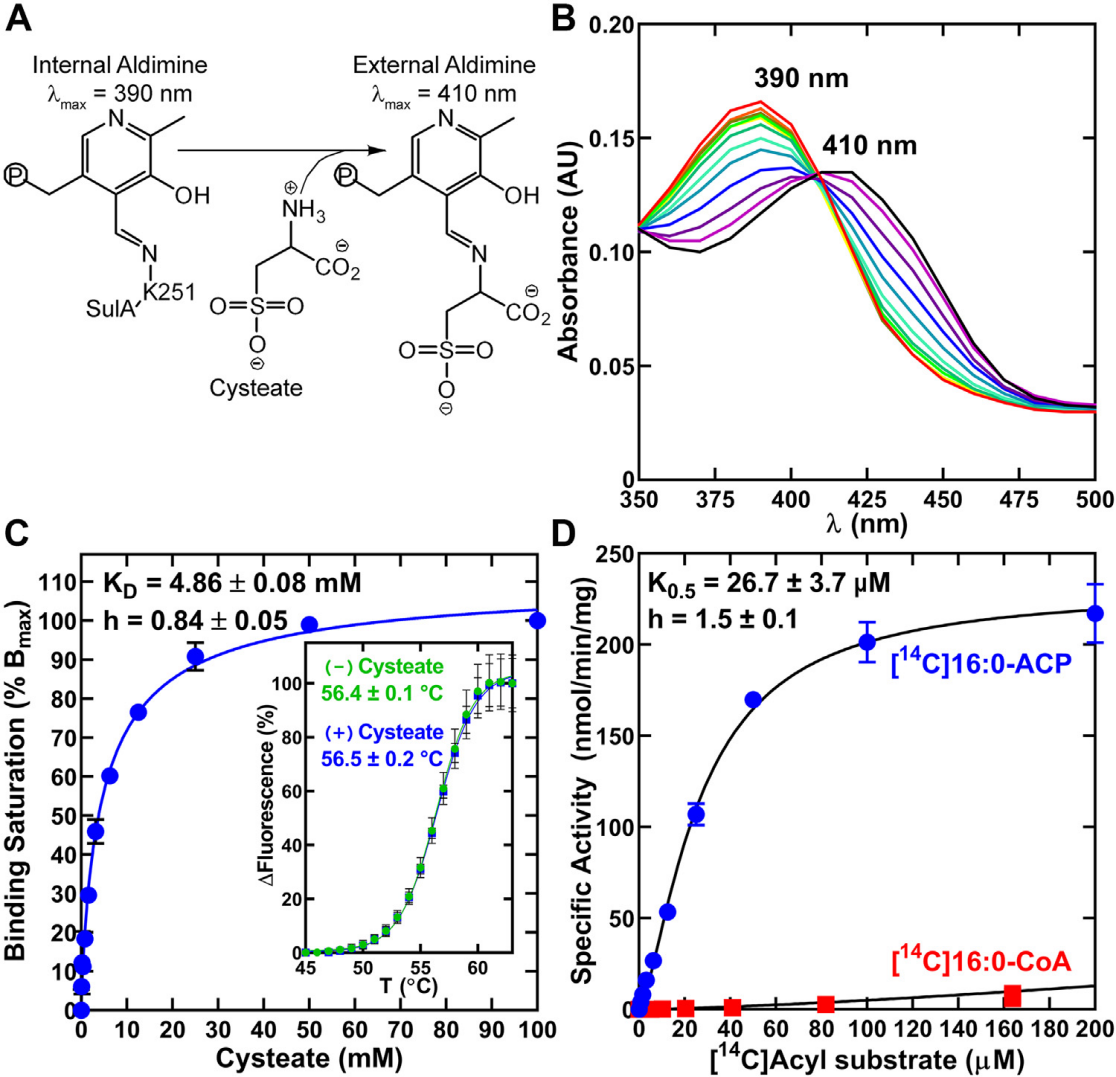
**SulA prefers acyl-ACP as substrate.***A*, schematic of the cysteate-dependent transition between internal (A_max_ = 390 nm) and external (A_max_ = 410 nm) forms of the PLP aldimine. *B*, cysteate titration (0, 0.097.7, 0.1953, 0.3906, 0.7812, 1.6, 3.1, 6.3, 12.5, 25, 50, and 100 mM; plotted from *red* to *violet*) causes a spectral shift in SulA absorption spectrum. *C*, the data from three independent cysteate titrations were fit to the Hill equation (line) using GraphPad Prism software to calculate the K_D_ and Hill coefficient (h). *Inset*, protein thermal denaturation analysis shows cysteate binding does not alter the thermal stability of SulA. Points were fit to the Boltzmann equation (n = 3). *D*, specific activities of SulA calculated as a function of [^14^C]16:0-ACP (*blue*) or [^14^C]16:0-CoA (*red*). The data are from three independent experiments per acyl substrate, and the enzymatic behavior of SulA was fit to the Hill equation (*line*). ACP, acyl carrier protein; PLP, pyridoxal phosphate; SulA, cysteate acyl-ACP transferase.

The canonical mechanism for PLP participation in condensation reactions starts with PLP linked by a Schiff base to a conserved lysine residue as the internal aldimine (Fig. 4*A*). Following substrate binding, in this case cysteate, the covalent bond between PLP and the protein breaks and a new bond forms with the α-amino group of the substrate to form the external aldimine (Fig. 4*A*) (39). UV-Vis spectra distinguish between these two states (34, 39). The conversion of the SulA-PLP internal aldimine to the PLP-cysteate external aldimine was measured at increasing concentrations of cysteate, which resulted in a redshift in absorption maxima from 390 nm to 410 nm (Fig. 4*B*). Absorption differences were used to plot a binding isotherm and determine the apparent cysteate dissociation constant (K_D_) (Fig. 4*C*). The 4.86 ± 0.08 mM cysteate K_D_ measured for SulA is slightly higher than the 1.1 ± 0.1 mM serine K_D_ measured for Spt by this method (34). A Hill coefficient of h = 0.84 ± 0.05 indicates cysteate binding is not a cooperative process, and thermal denaturation analysis of the internal (0 mM cysteate) and external aldimines (50 mM cysteate) shows cysteate binding does not alter SulA thermal stability (Fig. 4*C*, *Inset*).

SulA activity was measured using increasing concentrations of [^14^C]16:0-ACP and 100 mM cysteate and TLC was used to separate 3-keto[^14^C]capnine from [^14^C]16:0-ACP. The formation of 3-keto[^14^C]capnine was measured and the kinetics fit into an allosteric sigmoidal model with a calculated K_0.5_ of 26.7 for ± 3.7 μM for [^14^C]16:0-ACP (Fig. 4*D*). A Hill coefficient of h = 1.5 ± 0.1 indicates the protomers exhibit positive cooperativity in acyl-ACP binding. [^14^C]16:0-CoA is a poor substrate for SulA (Fig. 4*D*) with an apparent K_0.5_ in the mM range (Fig. 4*D*).

### SulA X-ray structure

**Figure 5:**
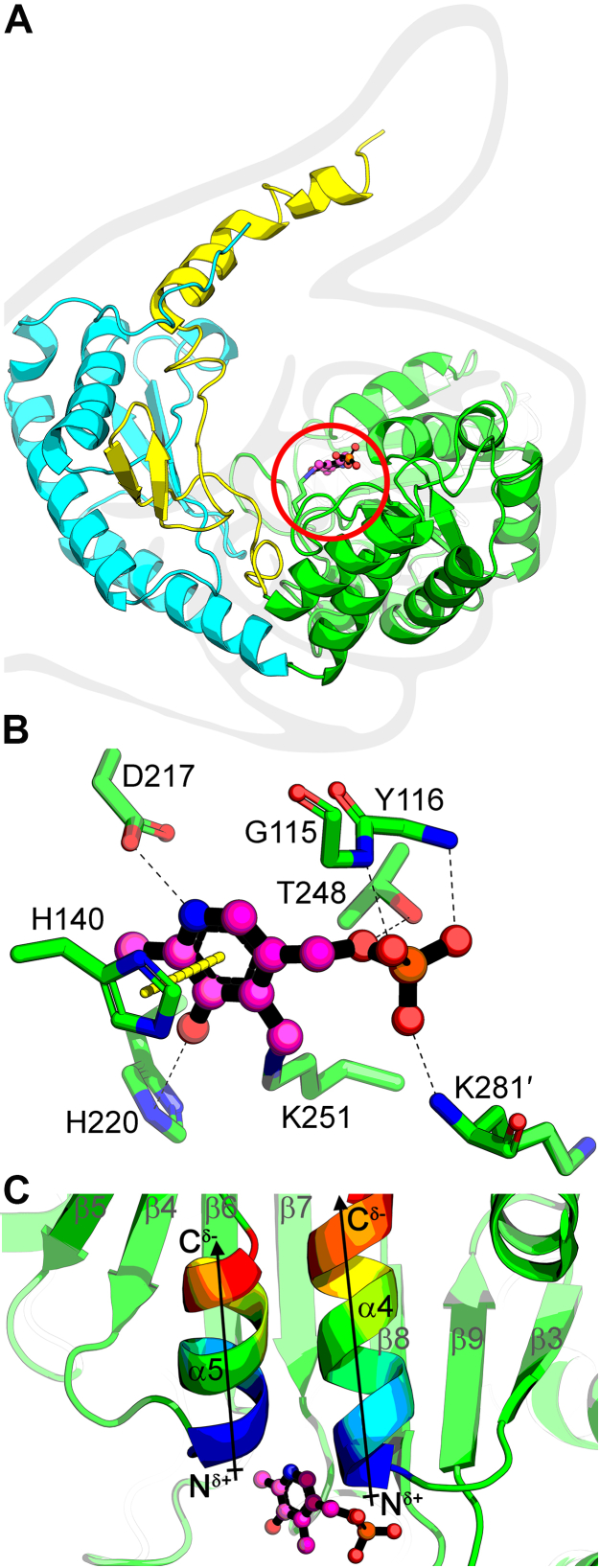
**SulA X-ray structure.***A*, the SulA monomer has a 3-domain architecture resembling an open fist with an extended thumb. The amino terminal “thumb” domain is *yellow*, internal core “fingers” domain is *green*, and carboxy terminal “hand base” is *cyan*. The catalytic Lys251 (*green stick*) is covalently bound to PLP (*magenta ball-and-stick*) in the active site palm (*red circle*). *B*, the hydrogen bonds (*black dashes*), a π-π stacking interaction (*thick yellow dash*), and covalent bond with residue Lys251 anchor PLP in the active site. Lys281′ is from the opposite protomer. *C*, the partial positive charges that arise from the orientation of α4 and α5 helix dipole “fingers” that anchor PLP in the active site are shown in rainbow from carboxy terminus negative (*red*) to amino terminus positive (*blue*). PLP, pyridoxal phosphate; SulA, cysteate acyl-ACP transferase.

The 2.9 Å SulA X-ray structure was determined and the data processing and structure refinement statistics are provided in Table 1. The histidine tag is not visible but the rest of the polypeptide sequence starting with SulA Met1 through the carboxy terminal Arg423 are resolved. There were six molecules in the asymmetric unit with an average R.M.S.D. of 0.4 Å. SulA protomers have high overall structural similarity to bacterial PLP-dependent Spt with an R.M.S.D. of 1.28 ± 0.07 Å compared to the Spt of *Sphingobacterium multivorum* (PDBID: 3A2B). The SulA protomer resembles an open fist with an extended thumb and contains three domains (Fig. 5*A*): an amino terminal domain extends from the ball to the top of the thumb (residues 1–60), a compact core domain represented by the bent fingers (residues 61–301), and a carboxy terminal domain that lies along the back of the hand (residues 302–423). A contiguous electron density is present across the internal aldimine complex and definitively identifies Lys251 as the PLP attachment site (Fig. S4).

**Table 1:** Data collection and model refinement statistics

Data collection	SeMet-SulA (7U7H)
Space group	*P*3_1_
Cell dimensions	
*a*, *b*, *c* (Å)	127.5, 127.5, 177.3
α, β, γ (°)	90, 90, 120
Resolution (Å)	29.55–2.90 (3.07–2.90)^a^
Unique reflections	142,317 (22,569)
*R*_merge_ (%)	11.5 (121.9)
CC_1/2_	99.8 (78.2)
*I/σ(I)*	11.9 (1.5)
Completeness (%)	99.6 (98.3)
Redundancy	5.5 (5.3)
Wilson B factor (Å^2^)	73.6
SHELXD: Data used (Å)	20–3.4
CC_all_/CC_weak_	44.6/16.0
Combined figure of merit (CFOM)	60.6
PAT figure of merit (FOM)	3.44
Number of Se atoms located	106 out of 114
Refinement	
Refinement resolution (Å)	28.95–2.90 (3.00–2.90)
Reflections used in refinement	71,215 (6999)
R_work_ (%)	21.8 (35.3)
R_free_ (%)^b^	26.8 (37.5)
No. of nonhydrogen atoms	18,499
Macromolecules	18,469
Ligands	30
Ramachandran statistics (%)	
Favored	94.6
Allowed	5.2
Outliers	0.2
RMSDs	
Bond lengths (Å)	0.006
Bond angles (°)	0.933
Clash score	15.1
Average B factors (Å^2^)	100.0
Macromolecules	100.1
Ligands	61.9

a Statistics for the highest-resolution shell are shown in parentheses.

b R_free_ test set uses ∼5% of the data.

PLP is bound to the central core domain that consists of a seven-stranded β-sheet with six parallel strands (β3, β4, β5, β6, β7, β8) and an antiparallel strand (β9) and is flanked by α-helices (Fig. 5*A*). This arrangement of mixed β-strands is an evolutionarily conserved topological feature of the fold-type I family of PLP-dependent enzymes (40). PLP is coordinated by the main chain nitrogen atoms of Gly115 and Tyr116 from the amino terminus of α4, the side chain hydroxyl group of Thr248, and the main chain nitrogen atom of Lys281′ from the opposite protomer (Fig. 5*B*). The seven stranded β-sheet (β3–β9) curves to cradle two α-helices (α4 and α5) that form finger-like helix dipoles that are oriented to contribute positive charge to the PLP-binding site (Fig. 5*C*). The α4 positive helix dipole participates in neutralizing the negative charge of the PLP phosphate and the positive α5 dipole (Fig. 5*C*) terminates with a parallel π-π stacking interaction between the side chain imidazole group of His140 and the pyridoxal aromatic ring of PLP (Fig. 5*B*). The PLP ring is also coordinated by the side chain carboxyl of Asp217 interacting with the pyridoxal nitrogen atom and the imidazole group of His220 and the pyridoxal hydroxyl group (Fig. 5*B*). The constellation of amino acid residues stabilizing PLP binding is conserved across AOS enzymes (Fig. S3) and the roles of residues His140, Asp217, and His220 in PLP binding are established in Spt (35). Their conservation and identical organization in the SulA PLP-binding residues show that SulA shares a common PLP-binding mode with other AOS enzymes.

**Figure 6:**
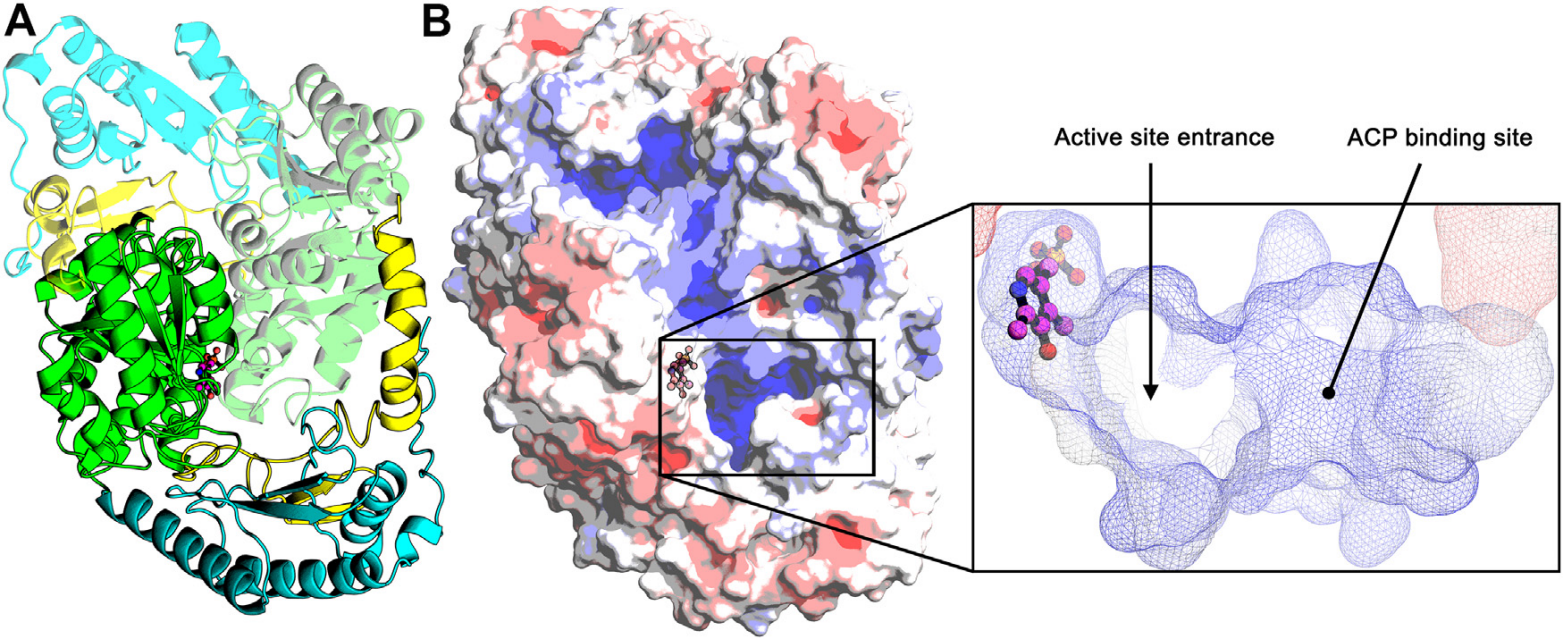
**Dimerization closes the active site and creates an ACP-binding surface.***A*, the open, solvent-exposed active site in the SulA protomer is enclosed by dimerization. Both protomers contribute to the surface that forms the active site and buries the PLP. *B*, the external surface created by both protomers creates an electropositive surface cleft adjacent to the opening to the active site. Electrostatic surface visualization is shown from −5 kT/e (*red*) to +5 kT/e (*blue*) of the SulA dimer solvent-accessible surface. The zoomed-in image shows the active site entrance adjacent to the electropositive ACP-binding patch. Calculated and displayed by APBS (63). ACP, acyl carrier protein; PLP, pyridoxal phosphate; SulA, cysteate acyl-ACP transferase.

The open SulA active site in the monomer is enclosed by dimerization. The palm of the hand contains the dimerization interface that is stabilized by interacting helices α2, α4, α5, and α8 of the two SulA core domains. Each protomer has a total surface area of ∼25,300 Å^2^, and ∼5500 Å^2^ (∼22%) is buried upon dimerization. These values are similar to what is reported for Spt (35). The interlocked SulA protomers are tightly packed in the homodimer and each domain contacts a portion of the opposite protomer (Fig. 6*A*). Helix α1 from the amino terminal domain is kinked, which allows it to lay on the surface of the compact core “fingers” of the opposite protomer of the dimer. Dimerization establishes the active site by forming a surface to enclose the active site and creates a hydrophobic cleft that leads to Lys251 in each protomer. Both protomers contribute positively charged residues that cluster at the entrance to the active site entrance. This surface feature is common to ACP-dependent enzymes (41) and suggests the ACP-binding site is located adjacent to the entrance of the PLP active site (Fig. 6*B*). The collaboration between the protomers to form this surface feature may underlie the cooperative ACP-binding behavior of SulA. ACP-binding sites are combinatorial; therefore, a site-directed mutagenesis campaign will be needed in future studies to validate the ACP-binding site and assess the individual contributions of positively charged amino acid side chains to ACP binding.

### Role of Lys281′

**Figure 7:**
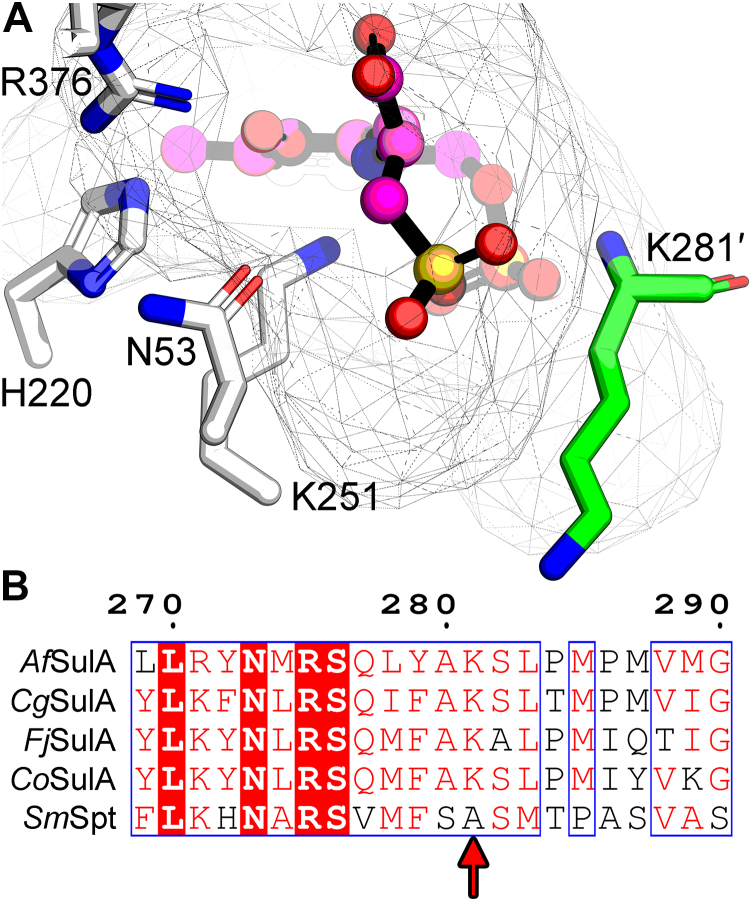
**The SulA active site.***A*, conserved residues within the SulA active site. The cysteate-PLP external aldimine (*magenta*) was docked into the empty SulA active site using Maestro (Schrödinger, LLC). The cysteate-PLP inferences in the crystal structure are based on docking. Residues common to SulA and to the AOS superfamily of enzymes are shown in *white* and labeled using SulA numbering. Lys281′ (*green*) is unique to SulA and forms one side of the active site. *B*, alignment of the amino acid sequences from *Alistipes finegoldii* SulA (*Af*SulA, Uniprot accession I3YKQ4), *Chryseobacterium gleum* SulA (*Cg*SulA, Uniprot accession A0A448AYE2), *Flavobacterium johnsoniae* SulA (*Fj*SulA, Uniprot accession A5FH75), *Capnocytophaga ochracea* SulA (*Co*SulA, Uniprot accession C7M8J6), and *Sphingobacterium multivorum* Spt (*Sm*Spt, Uniprot accession A7BFV6). The *red arrow* points to Lys281. AOS, α-oxoamine synthase; PLP, pyridoxal phosphate; Spt, serine palmitoyltransferase; SulA, cysteate acyl-ACP transferase.

An energy minimized docking solution placed the cysteate external aldimine in SulA with the cysteate sulfono side chain in the equivalent position as the serine side chain hydroxyl in *S. multivorum* Spt (*Sm*Spt; PDBID: 3A2B) PLP-serine external aldimine complex (*Sm*Spt) (Fig. 7*A*). The negatively charged sulfono group lies between Lys251, which becomes liberated when the PLP-cysteate aldimine is formed and Lys281′ from the opposite protomer. Arg376, His140, Asn53, and Lys251 in SulA are all conserved in the Spt enzyme superfamily (Fig. S3) and located in the same positions in the active sites of SulA and *Sm*Spt (Fig. 7*A*). There is only one clear difference between the *Sm*Spt and SulA active sites. In SulA, and the other cysteate acyltransferases, Lys281 is present in place of the alanine residue found in bacterial Spt (Fig. 7*B*). The backbone amides of the Spt alanine and SulA Lys281 both hydrogen bond with the PLP phosphate. Lys281 is located in a position to potentially promote cysteate binding by neutralizing the charged sulfonate group in the active site, or it may have a structural role in the formation and integrity of the SulA active site.

**Figure 8:**
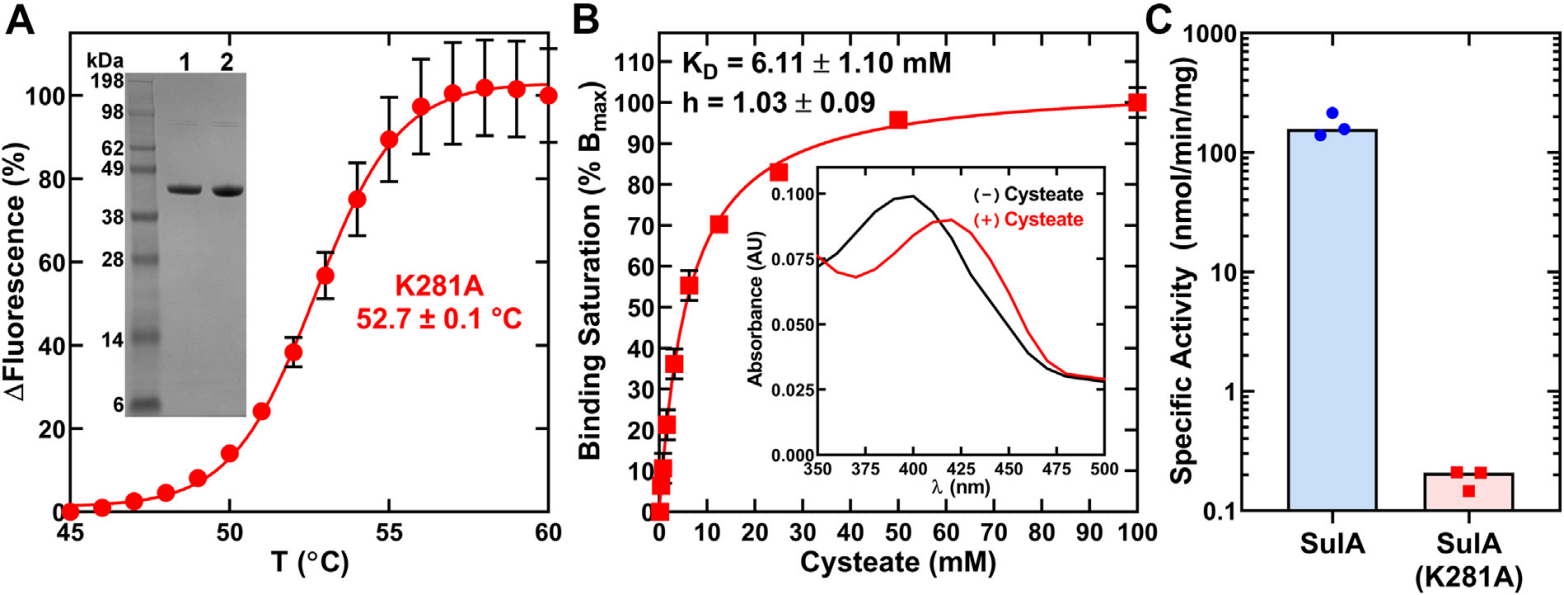
**Analysis of SulA(K281A).***A*, the thermal denaturation profile of SulA(K281A) (n = 3). *Inset*, purified SulA (lane 1) and SulA(K281A) (lane 2). *B*, the binding of cysteate to SulA(K281A) (n = 3). *Inset*, the absorption spectra of SulA(K281A) with 100 mM cysteate (+) or without cysteate (−). *C*, specific activities of SulA and SulA(K281A) (n = 3). SulA, cysteate acyl-ACP transferase.

We constructed the SulA(K281A) mutant, and pure SulA(K281A) is a folded, stable protein (Fig. 8*A*). A cysteate titration experiment shows SulA(K281A) bound cysteate and formed the PLP-cysteate external aldimine normally (Fig. 8*B*). SulA(K281A) has the same absorption spectra for the internal and external aldimine as SulA (Fig. 8*B*, *Inset*). Although these first steps in the catalytic cycle are intact in SulA(K281A), the mutant is three orders of magnitude less active than SulA (Fig. 8*C*). These data support the conclusion that Lys281 has a structural role in assembling the SulA active site rather than promoting cysteate binding by charge neutralization.

## Discussion

**Figure 9:**
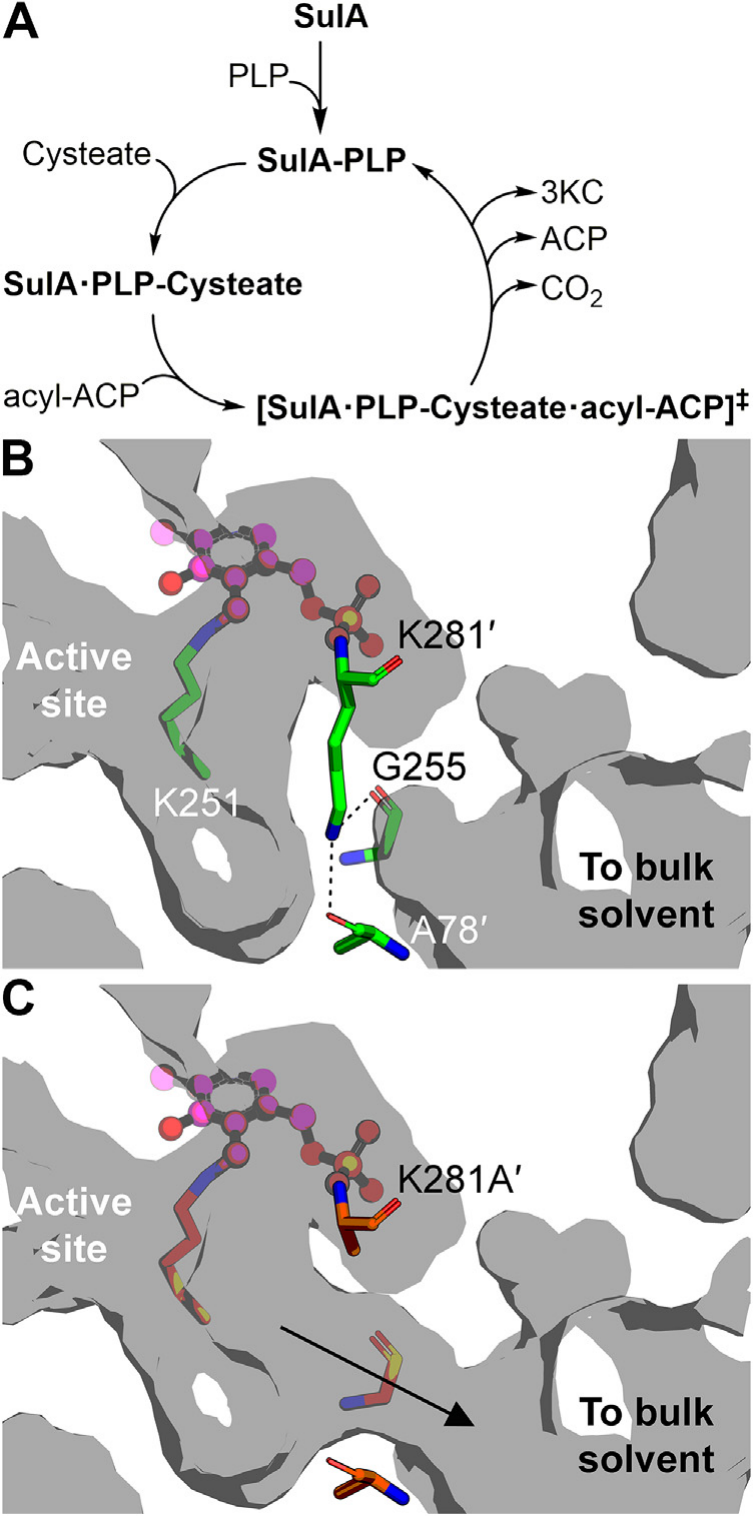
**SulA catalysis.***A*, the SulA catalytic cycle is proposed to be the same as for Spt. The first step is the formation of the SulA internal aldimine with Lys251. Next, cysteate binds to the enzyme and the internal aldimine converts to the external cysteate aldimine. Acyl-ACP binds next to form the catalytically competent ternary complex (^‡^) and the transition state breaks down to release ACP, CO_2_, and 3-ketocapnine (3KC), coupled with the reformation of the SulA internal aldimine. *B*, the side chain of Lys281′ forms one side of the active site cavity (*gray*) and is fixed in position by hydrogen bonds with the backbone carbonyls of Ala78′ and Gly255. The Lys281 carbons form a surface that encloses the active site. *C*, the SulA(K281A) mutation creates a hole in the active site surface that opens the site to bulk solvent. SulA and SulA(K281A) interior surface was rendered using PyMOL. ACP, acyl carrier protein; Spt, serine palmitoyltransferase; SulA, cysteate acyl-ACP transferase.

This work identifies the gene, enzyme, substrates, and product of the first step in sulfonolipid biosynthesis in *A. finegoldii*, an anaerobic gut commensal. The SulA reaction mechanism is essentially the same as described for Spt (Fig. 9*A*). The first step is the formation of the SulA PLP internal aldimine with Lys251. SulA is not fully in the PLP internal aldimine state when purified due to insufficient PLP available in the *E. coli* expression system. SulA must be loaded with PLP to create the fully active enzyme. Cysteate binding triggers the formation of an external aldimine of PLP with cysteate that is evidenced by the redshift in the UV-Vis spectra of the SulA·PLP·cysteate complex. Acyl-ACP is the next substrate to bind. Previous work with bacterial Spt (35, 36, 37) and SulA (29, 30) used acyl-CoA to characterize the enzymes. Acyl-CoA is the correct substrate for the mammalian Spt, but it is likely that acyl-ACP derived from the *de novo* fatty acid biosynthetic pathway are the preferred acyl donors for bacterial Spt and SulA. In the case of *A. finegoldii*, CoA thioesters do not participate in lipid metabolism (8) and it is likely that other Bacteroidetes are similarly wired. The source of the 3-hydroxy-fatty acids that are used in the formation of sulfobactin A (Fig. 1*A*) is explained by the acyltransferase using 3-hydroxy-acyl-ACP derived from fatty acid synthesis in the same manner as the LpxA acyltransferase extracts 3-hydroxy-acyl-ACP for lipid A biosynthesis from the fatty acid pathway (42). The external aldimine then condenses with the incoming acyl-ACP to release CO_2_, ACP, and 3-ketocapnine. The PLP internal aldimine is reformed in this last step to return the enzyme to its initial state ready to bind another cysteate (Fig. 8*A*). The SulA catalytic cycle is identical in steps and chemistry to that proposed for Spt.

The distinguishing feature of SulA is the presence of Lys281′ in the active site instead of an alanine residue found in the equivalent position in the Spt enzyme superfamily. There are four conserved residues (SulA numbering: Arg376, His140, Asn53, and Lys251) in the Spt enzyme family (Fig. S3) that are located at the exact same 3-dimensional positions in the SulA and Spt active sites (Fig. 7*A*). Their roles in Spt catalysis are known. The side chain guanidinium of Arg376 and side chain carboxamide of Asn53 are thought to activate the carboxylate of the amino acid substrate as a leaving group (36, 43). Some Spt enzymes contain a Tyr in the Asn53 position (Fig. S5). The π-π stacking interaction between the His140 side chain and the PLP ring is maintained in the external aldimine, but when acyl-ACP binds to Spt, His140 reorients to a position perpendicular to the PLP ring system (36, 44). In this conformation, His140 interacts with the thioester carbonyl of acyl-ACP to stabilize the negative charge that develops on this oxygen in the condensation transition state (36, 44). It is proposed that when the Spt external aldimine is formed, the liberated Schiff base lysine (Lys251 in SulA) rotates to hydrogen bond with the serine substrate hydroxyl side chain (34). This role for the Schiff base lysine has also been proposed for other AOS enzymes (39, 45, 46, 47), and by analogy, SulA Lys251 would neutralize the negative charge on the sulfonic acid in the external aldimine. Lys281 is an alanine in Spt, and in both enzymes, the backbone amide forms a hydrogen bond with the phosphate of PLP. In SulA, the carbon atoms of the Lys281 side chain have a structural role in forming one side of the active site enclosure (Fig. 9*B*). The absence of this side chain in SulA(K281A) creates a hole in the active site that opens it to bulk solvent (Fig. 9*C*). We conclude that the inability to enclose the active site in SulA(K281A) is the reason that the otherwise properly folded and functional mutant protein is catalytically inactive. Lys281 is conserved in known SulA enzymes and may be used to distinguish sulfonolipid and sphingolipid-condensing enzymes in bacteria. How SulA achieves cysteate selectivity remain an open question. The SulA active site contains alanine side chains at positions occupied by amino acids with bulkier side chains in Spt (Fig. S5). This difference creates a larger pocket to accommodate the bulkier substrate cysteate sulfonic acid than the Spt pocket. The Lys281 side chain could participate in neutralizing the sulfonic acid given its proximity to the substrate, but this interaction would require a conformation of the Lys281 side chain that is unavailable in the docking simulation. Therefore, a structure of the SulA external aldimine is needed to better understand the SulA substrate selectivity for cysteate.

## Experimental procedures

### Materials

All chemicals and reagents were obtained from Sigma-Aldrich or Fisher Scientific unless otherwise indicated.

### Molecular biology

*A. finegoldii* genes *alfi_1465* (Uniprot ID: I3YLE0) and *alfi_1224* (Uniprot ID: I3YKQ4) DNA sequences were optimized for gene expression in *E. coli* using GeneArt Gene Synthesis Technology (Life Technologies) and synthesized with 5′-NdeI and 3′-EcoRI restriction sites for cloning into the pPJ131 vector for constitutive expression in *E. coli* (48). For protein purification, the *alfi_1224* construct was cloned into the pET28b plasmid to make pET-SulA using the same restriction sites to introduce an amino-terminal His_6_-tag. The pET-SulA(K281A) plasmid was constructed by modifying pET-SulA using the QuikChange Lightning site-directed mutagenesis kit (Agilent Technologies) and primers Alfi1224-K281A-F, 5′- gttcccaattgtacgcagcgtccctgccaatgccga and Alfi1224-K281A-R, 5′-tcggcattggcagggacgctgcgtacaattgggaac.

### Preparation of acyl-ACP

ACP was prepared by incubating 750 μM apo-ACP with 50 mM Tris, pH 8.5, 10 mM MgCl_2_, 1 mM DTT, 4 μM His-tagged *E. coli* AcpS ([ACP]synthase), and 4 mM CoA in a 1 ml reaction at 37 °C for 2 h, as described previously (49). The His-tagged AcpS was removed by adding nickel agarose beads (Gold Biotechnology) to the reaction, incubating the mixture at room temperature for 10 min, and then excluding the His-tagged AcpS-linked nickel agarose beads from the mixture by gel filtration using a PD-10 column (GE Healthcare). ACP was eluted into 20 mM Tris, pH 7.5.

16:0-ACP was prepared by incubating 150 μM ACP with 100 mM Tris, pH 8.0, 10 mM ATP, 20 mM MgCl_2_, 2 mM DTT, 5 μM His-tagged *Ct*Aas (acyl-ACP synthetase from *Chlamydia trachomatis*), and 200 μM 16:0 in a 1 ml reaction at 37 °C for 2 h, as described (50). [^14^C]16:0-ACP was prepared by incubating 150 μM ACP with 100 mM Tris, pH 8.0, 10 mM ATP, 20 mM MgCl_2_, 2 mM DTT, 5 μM His-tagged *Ct*Aas, 140 μM 16:0, and 60 μM [^14^C]16:0 (PerkinElmer, Inc) in a 1 ml reaction at 37 °C for 2 h. The His-tagged *Ct*Aas was removed by adding nickel agarose beads (Gold Biotechnology) to both reactions, incubating the mixtures at room temperature for 10 min, and then excluding the His-tagged *Ct*Aas-linked nickel agarose beads from the mixtures by gel filtration using PD-10 columns (GE Healthcare). The 16:0-ACP or [^14^C]16:0-ACP were eluted into 20 mM Tris, pH 7.5. Conversion of apo-ACP to ACP and then acyl-ACP was confirmed by 2.5 M urea, 15% w/v acrylamide native gel electrophoresis (Fig. S2), and radioactivity was quantified by scintillation counting.

### Lysate assay for SulA activity

Expression plasmids containing nothing (pPJ131), *alfi_1465* (p*Af*1465), or SulA (pSulA) were transformed into *E. coli* strain Top10 cells (Invitrogen), and these strains were grown in 100 ml terrific broth medium at 37 °C and 200 rpm. Cells were harvested by centrifugation (10,000*g* for 10 min) at A_600_ = 4.0 and the cell pellets were washed in PBS, then H_2_O, and then resuspended in 1 ml of 150 mM NaCl, 100 mM Tris, pH 7.2. Cells were broken by two passes through a French press at a working pressure of 20,000 psi, and cell free bacterial lysates were separated from cellular debris by centrifugation (20,000*g* for 30 min). The protein concentration of each lysate was determined by Coomassie Protein Assay (Thermo Scientific). SulA activity assays were carried out at 30 °C for 30 min in 20 μl reactions containing 500 ng lysate, 200 μM [^14^C]16:0-ACP, 1% Triton X-100, 100 mM potassium phosphate buffer, 150 mM NaCl, pH 7.6, with and without 20 mM cysteate. The entire reaction was spotted on a Silica Gel H thin-layer plate (Spectrum Chemical) and separated using ethanol:chloroform:triethylamine:H_2_O (34:30:35:6.5, v/v). Product formation was visualized using a Typhoon PhosphorImager and quantified using ImageQuant (Cytiva Life Sciences).

### Preparation of SulA, SulA(K281A), and SeMet-SulA

*E. coli* strain BL21(DE3) cells harboring the pET-SulA or pET-SulA(K281A) expression plasmids were grown in 2 l LB medium with 50 μg/ml kanamycin (Gold Biotechnology) at 37 °C and 200 rpm shaking to A_600_ = 0.6. The culture was cooled to 16 °C and then SulA expression was induced with 1 mM IPTG overnight. Cells were harvested by centrifugation (10,000*g* for 10 min) and resuspended in 200 mM NaCl, 10 mM imidazole, 20 mM Tris, pH 8.0. Cells were broken by two passages through a cell disruptor and SulA or SulA(K281A) were separated from cell lysates by nickel agarose beads (Gold Biotechnology). Pure SulA proteins were eluted from the nickel agarose beads with 200 mM NaCl, 250 mM imidazole, 20 mM Tris, pH 8.0. The eluant was gel filtered into a buffer containing 200 mM NaCl, 20 mM Tris, pH 8.0 using a HiLoad 16/600 Superdex 200 column (Cytiva Life Sciences). The molecular weight was calculated by a standard curve using ferritin (440 kDa), aldolase (158 kDa), conalbumin (75 kDa), ovalbumin (44 kDa), carbonic anhydrase (29 kDa), ribonuclease A (13.7 kDa), and aprotinin (6.5 kDa). SulA protein labeled with selenomethionine (SeMet-SulA) was prepared by transforming *E. coli* strain B834(DE3) (an *E. coli* methionine auxotroph from Novagen) with pET-SulA plasmid and growing the cells in 200 ml Luria broth with 50 μg/ml kanamycin (Gold Biotechnology) at 37 °C and 200 rpm shaking to stationary phase. The B834(DE3) cells harboring pET-SulA were harvested by centrifugation (10,000*g* for 10 min) and washed 3× in 20 mM Tris, 200 mM NaCl, pH 8.0. Cells were resuspended in 5 ml of the same buffer and added to 4 l of selenomethionine medium (Molecular Dimensions) containing 50 μg/ml kanamycin (Gold Biotechnology) and 42.5 mg/l selenomethionine. Cells were grown at 37 °C and 200 rpm shaking until A_600_ = 0.6 at which point the culture was cooled to 16 °C and SulA expression was induced with 1 mM IPTG overnight. Cells were harvested by centrifugation (10,000*g* for 10 min) and resuspended in 200 mM NaCl, 10 mM imidazole, 20 mM Tris, pH 8.0, and the same purification strategy was used to purify SeMet-SulA. SulA was dialyzed at 4 °C against a buffer containing 250 μM PLP (TCI America), 150 mM NaCl, 20 mM potassium phosphate buffer, pH 7.6, to optimize PLP incorporation and the excess PLP was removed by dialysis at 4 °C against a buffer containing 150 mM NaCl, 20 mM potassium phosphate buffer, pH 7.5. Multiple batches of protein were made for this study and the data collected are the same between them and used interchangeably.

### Analytical ultracentrifugation

Sedimentation velocity experiments were conducted in a ProteomeLab XL-I analytical ultracentrifuge (Backman Coulter) following standard protocols (51, 52). Samples in buffer containing 150 mM NaCl, 1 mM DTT, 20 mM potassium phosphate buffer, pH 7.5 were loaded into cell assemblies comprised of double sector charcoal-filled centerpieces with a 12 mm path length and sapphire windows. Buffer density and viscosity were calculated using the software SEDNTERP (http://www.jphilo.mailway.com/download.htm). The partial specific volume and molecular mass of SulA was calculated from the amino acid sequence in SEDFIT (https://sedfitsedphat.nibib.nih.gov/software/default.aspx). The call assemblies, containing identical sample and reference buffer volumes of 390 μl, were placed in a rotor and temperature equilibrated at rest at 20 °C for 2 h before it was accelerated from 0 to 50,000 rpm. Rayleigh interference optical data were collected at 1 min intervals for 12 h. The velocity data were modeled with diffusion-deconvoluted sedimentation coefficient distributions *c*(*s*) in SEDFIT, using algebraic noise decomposition and with signal-average frictional ratio and meniscus position refined with nonlinear regression (53). The *s*-values were corrected for time and finite acceleration of the rotor was accounted for in the evaluation of Lamm equation solutions (54). Maximum entropy regularization was applied at a confidence level of *p* = 0.68.

### Cysteate binding and thermal stability

UV-Vis spectra were measured from 100 μl samples in a UV half area 96-well plate (Corning) using a Synergy H1 microplate reader (BioTek). Samples contained 150 mM NaCl, 20 mM potassium phosphate buffer, pH 7.5, 10 μM SulA (or SulA(K281A)), and either 0 mM, 97.7 μM, 195.3 μM, 390.6 μM, 781.2 μM, 1.6 mM, 3.1 mM, 6.3 mM, 12.5 mM, 25 mM, 50 mM, or 100 mM cysteate. Samples incubated for 30 min at room temperature before reading. The change in A_430_ was chosen to model cysteate binding because the Michaelis–Menten model gave the best fit at this wavelength and the data normalized to the maximal binding.

The thermal stability of the proteins was analyzed in the presence of 0 mM and 100 mM cysteate. Samples were added to the wells of ThermoGrid optically clear PCR places (Denville Scientific) and Sypro Orange Dye was added to a final concentration of 2.5×. The plates were centrifuged at 1000*g* for 5 min and then analyzed by the ABI 7300 real-time PCR system as described previously (48). Each sample was replicated three times, and the thermal melting point of each replicate was determined independently by fitting the data points to the Boltzmann equation using Graph-Pad software.

### Mass spectrometry analysis of the SulA product

Reactions containing 150 mM NaCl, 20 mM potassium phosphate buffer, 1% Triton X-100, 20 mM cysteate, pH 7.5, 100 nM SulA, 50 μM 16:0-ACP in 100 μl were incubated at 30 °C for 30 min. Methanol (80% final, v/v) was added to stop the reaction and the mixture was centrifuged at 20,000*g* for 10 min. The methanol extract supernatant was analyzed with a Shimadzu Prominence UFLC attached to a QTrap 4500 equipped with a Turbo V ion source (Sciex, Inc) looking for an *m/z* of 362.2 which corresponds to the product of the reaction. Samples were injected onto an XSelect HSS C18, 2.5 μm, 3.0 × 150 mm column (Waters) at 45 °C using a flow rate of 0.4 ml/min. Solvent A was water, and solvent B was acetonitrile. The HPLC program was the following: starting solvent mixture of 60% A/40% B, 0 to 1 min isocratic with 40% B; 1 to 16 min linear gradient 100% B; 16 to 25 min isocratic with 100% B; 25 to 27 min linear gradient to 40% B; 27 to 31 min isocratic with 40% B. The QTrap 4500 was operated in the negative mode for a product scan of m/z of 362.2 by direct injection. The ion source parameters were as follows: ion spray voltage, −4500 V; curtain gas, 25 psi; temperature, 270 °C; collision gas, medium; ion source gas 1, 20 psi; ion source gas 2, 35 psi; and declustering potential, −35 V. The system was controlled by the Analyst software (Sciex, Inc).

### Enzyme assay for SulA activity

SulA enzymatic activity was determined by measuring the conversion of [^14^C]16:0-ACP to 3-keto[^14^C]capnine by TLC. Enzyme reactions containing 150 mM NaCl, 100 mM potassium phosphate buffer, 1% Triton X-100, 100 mM cysteate, pH 7.0, 100 nM SulA in 20 μl were incubated for 20 min at 30 °C. The apparent K_0.5_ of [^14^C]16:0-ACP was determined by adding 0, 0.8, 1.6, 3.1, 6.3, 12.5, 25, 50, 100, or 200 μM [^14^C]16:0-ACP. Acyl-CoA assays used 0, 2.6, 5.1, 10.2, 20.5, 40.9, 81.9, 163.8, 327.5, 655, or 1310 μM [^14^C]16:0-CoA (American Radiolabeled Chemicals, Inc). The entire reaction was spotted on a Silica Gel H thin-layer plate (Spectrum Chemical) and separated using chloroform:methanol:ammonium hydroxide (40:10:1, v/v). 3-Keto[^14^C]capnine product formation was visualized using a Typhoon PhosphorImager and quantified using ImageQuant (Cytiva Life Sciences).

SulA substrate selectivity was determined from 20 μl enzyme reactions containing 150 mM NaCl, 100 mM potassium phosphate buffer, 1% Triton X-100, pH 7.0, 200 μM [^14^C]16:0-ACP, 100 nM SulA, and either 30 mM serine, 30 mM phosphoserine, or 30 mM cysteate. SulA and SulA(K281A) specific activities were compared in reactions containing 30, 100, or 200 mM cysteate. The reactions incubated for 20 min at 30 °C and then were spotted on a Silica Gel H thin-layer plate (Spectrum Chemical) and separated using ethanol:chloroform:triethylamine:H_2_O (34:30:35:6.5, v/v). 3-Keto[^14^C]capnine product formation was visualized using a Typhoon PhosphorImager.

### Crystallization and structure determination

The SeMet-SulA protein was concentrated to 27 mg/ml for crystallization. Initial screening was performed at 20 °C against the Protein Complex Suite (NeXtal Biotechnologies) by hanging-drop vapor diffusion method combining 200 nl protein and 200 nl precipitant. Diffraction quality crystals were obtained by combining 1.5 μl SeMet-SulA and 1.5 μl 12% w/v PEG8000, 100 mM sodium cacodylate, 100 mM calcium acetate, pH 5.0 to 5.5. Crystals were cryo-protected with 15% w/v PEG8000, 100 mM sodium cacodylate, 100 mM calcium acetate, pH 5.5, 30% v/v glycerol, and then flash frozen in liquid nitrogen for X-ray diffraction experiments. SeMet-SulA crystals were of better quality and diffracted to a higher resolution than the native SulA crystals. Se-SAD data were collected at the FMX 17-ID-2 beamline at NSLS-II to 2.9 Å and processed using XDS (55). SAD phasing was pursued using hkl2map (56); however, MR using a model (PDBID: 3A2B template with the N-terminal 40 residues truncated) generated by SWISS-MODEL (57) produced a more interpretable starting map. Six protomers were found in the asymmetric unit and the experimental phases were used during the initial stages of refinement. Model building was done using COOT (58), Se atom sites confirmed the sequence registry of the amino terminus, and iterative rounds of refinement were done using REFMAC (59) and phenix.refine (60). The refinement was monitored by following the R_free_ value calculated from a random subset (5%) of omitted reflections. The figures related to the protein structure were generated with PyMOL (61) using molecules A and B. Surface areas were calculated in PyMOL as described previously (62). The buried surface area by dimerization was calculated by subtracting the surface area of the homodimer from the combined surface area of the two individual protomers.

### Docking cysteate-PLP into the SulA active site

PLP was removed from the SulA dimer formed by molecules A and B in the crystal structure to make the receptor for the docking experiment. Docking was conducted in Maestro software (Schrödinger LLC). The SulA dimer was processed and minimized to prepare for docking using the Maestro Protein Preparation Wizard. The pKa and protonation states for the active site amino acids at pH 7 was predicted using the Epik tool within the Protein Preparation Wizard. The cysteate-PLP external aldimine was prepared using the Maestro LigPrep module, and the active site was prepared for docking using the Receptor Grid Generation module. The active site residue Lys251 that forms a Schiff base with PLP was defined as the receptor box center. Induced fit docking of cysteate-PLP into the SulA dimer was conducted using the Glide module. The solution shown in Figure 7 was the highest scoring solution that maintained protein–ligand interactions known to occur in the external aldimine state (*i.e.*, π-π stacking and hydrogen bond interactions with PLP-binding residues).

### Statistical analysis

All statistical analyses (*i.e.*, standard error, K_0.5_) and mathematical modeling (*i.e.*, Hill and Boltzmann equations) were performed using GraphPad Prism software version 9.1.1.

## Data availability

Coordinates and structure factors for the SeMet-SulA crystal structure have been deposited in the Protein Data Bank under accession code PDBID: 7U7H. All remaining data are contained within the article.

## Supporting information

This article contains supporting information (63).

## Conflict of interest

The authors declare that they have no conflicts of interest with the contents of this article.
