# Supplementary material for: Biochemical characterization of the first step in sulfonolipid biosynthesis in Alistipes finegoldii
Source: J Biol Chem. 2022 Jun 25;298(8):102195. doi: 10.1016/j.jbc.2022.102195 (PMC9304779; doi:10.1016/j.jbc.2022.102195)
Supplement: Supplemental Figures S1–S5 [file mmc1.pdf]

Supplemental Materials

Biochemical characterization of the first step in sulfonolipid biosynthesis in *Alistipes finegoldii*

Christopher D. Radka, Darcie J. Miller, Matthew W. Frank, Charles O. Rock

Figures S1-S5.

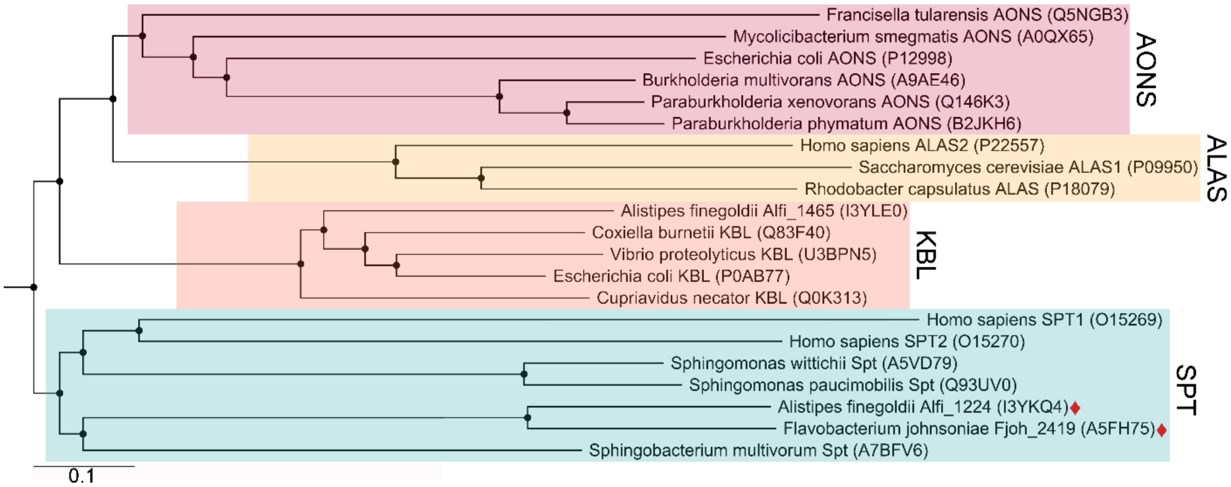

Figure S1. Evolutionary relationship between AOS family members. The AOS family has four main groups that diverged early in evolution. AONS, 8-amino-7-oxononanoate synthase; ALAS, aminolevulinic acid synthase; KBL, 2-amino-3-ketobutrate coenzyme A ligase; SPT, serine palmitoyltransferase. Red diamonds indicate that genes encoding SulA (*alfi_1224* and *fjoh_2419*) lie within the SPT group. The Uniprot accession code for each sequence is shown in parenthesis, and the phylogenetic tree was constructed in CLC Main Workbench (version 20.0).

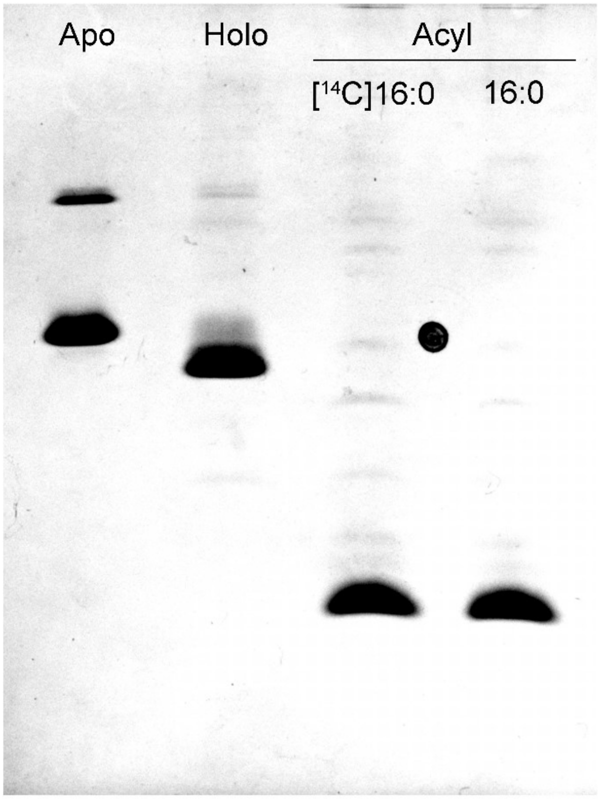

Figure S2. Acyl-ACP and [^{14}C]acyl-ACP synthesis. Conversion of apo-ACP to ACP and then to acyl-ACP was confirmed by 2.5 M urea, 15% w/v acrylamide native gel electrophoresis.

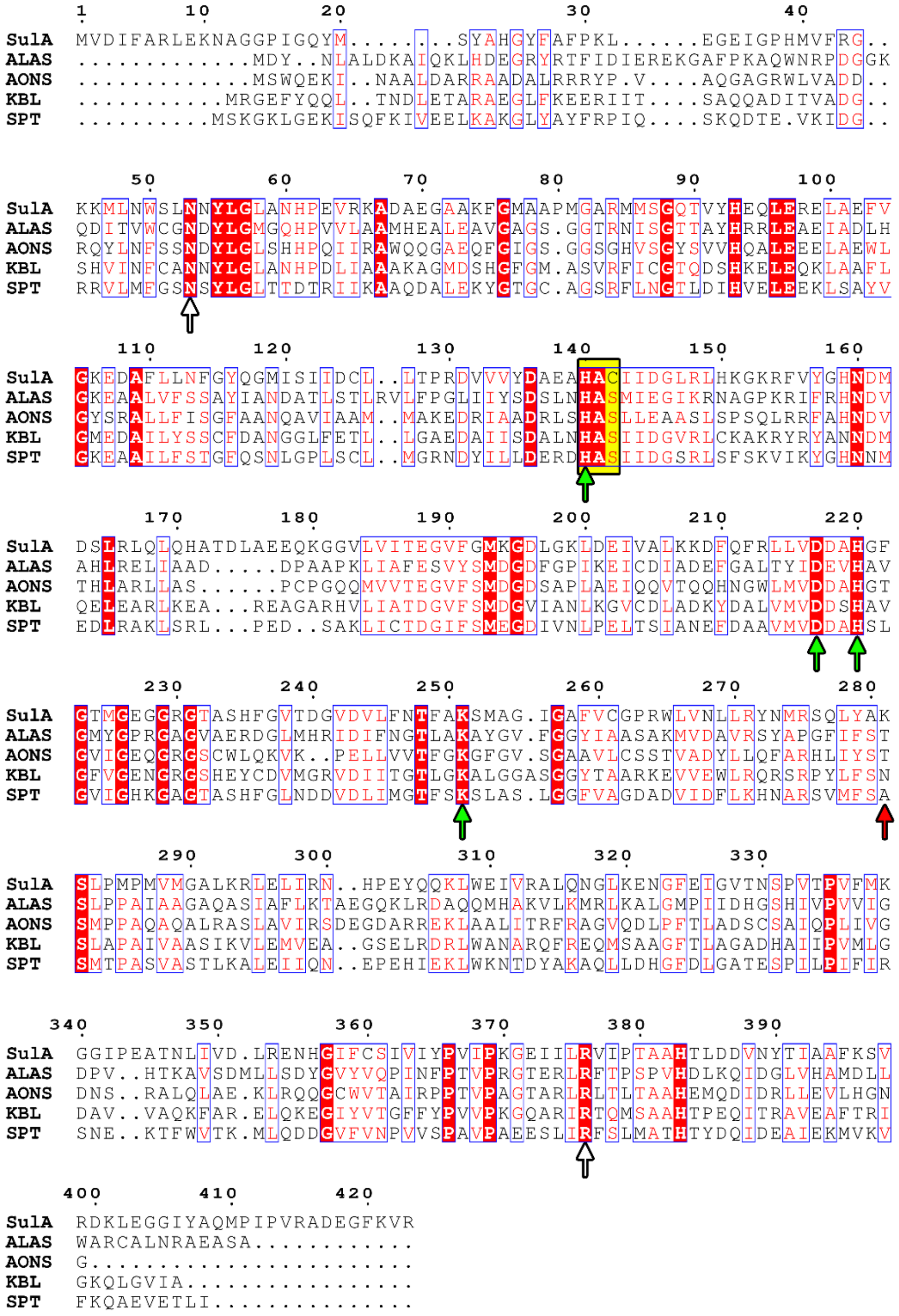

Figure S3. PLP binding sites in the AOS enzyme superfamily. Alignment of the SulA amino acid sequence versus sequences from structurally confirmed AOS enzymes deposited in the Protein Data Bank for serine palmitoyltransferase (SPT; PDBID: 3A2B), 8-amino-7-oxononanoate synthase (AONS; PDBID: 1DJE), 5-aminolevulinate synthase (ALAS; PDBID: 2BWN), and 2-amino-3-ketobutyrate CoA ligase (KBL; PDBID: 1FC4). Conserved PLP-binding residues are identified by green arrows, the conserved HAS/C PLP-binding motif is identified by yellow highlight, the white arrows indicate conserved active site residues that are implicated in the condensation reaction and the SulA-specific Lys281 is identified by the red arrow. Amino acid sequences were aligned using Clustal Omega (https://www.ebi.ac.uk/Tools/msa/clustalo/) and the ESPript program (http://endscript.ibcp.fr/ESPript/ESPript/index.php) was used to visualize sequence conservation. Sequence numbering is assigned to SulA.

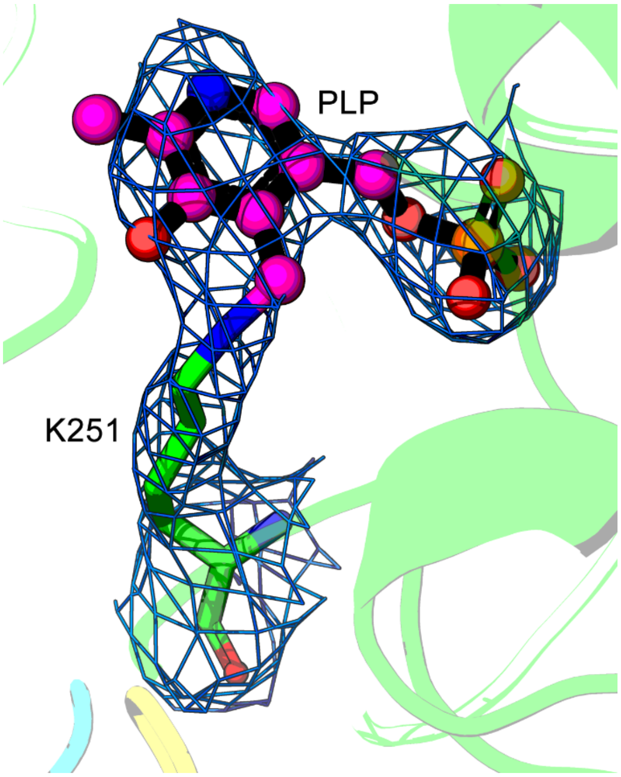

Figure S4. Contiguous electron density of the covalent internal aldimine complex. Electron density calculated from a $2mF_O–DF_C$ map contoured at 1 σ (blue mesh) shows contiguous electron density across PLP and Lys251.

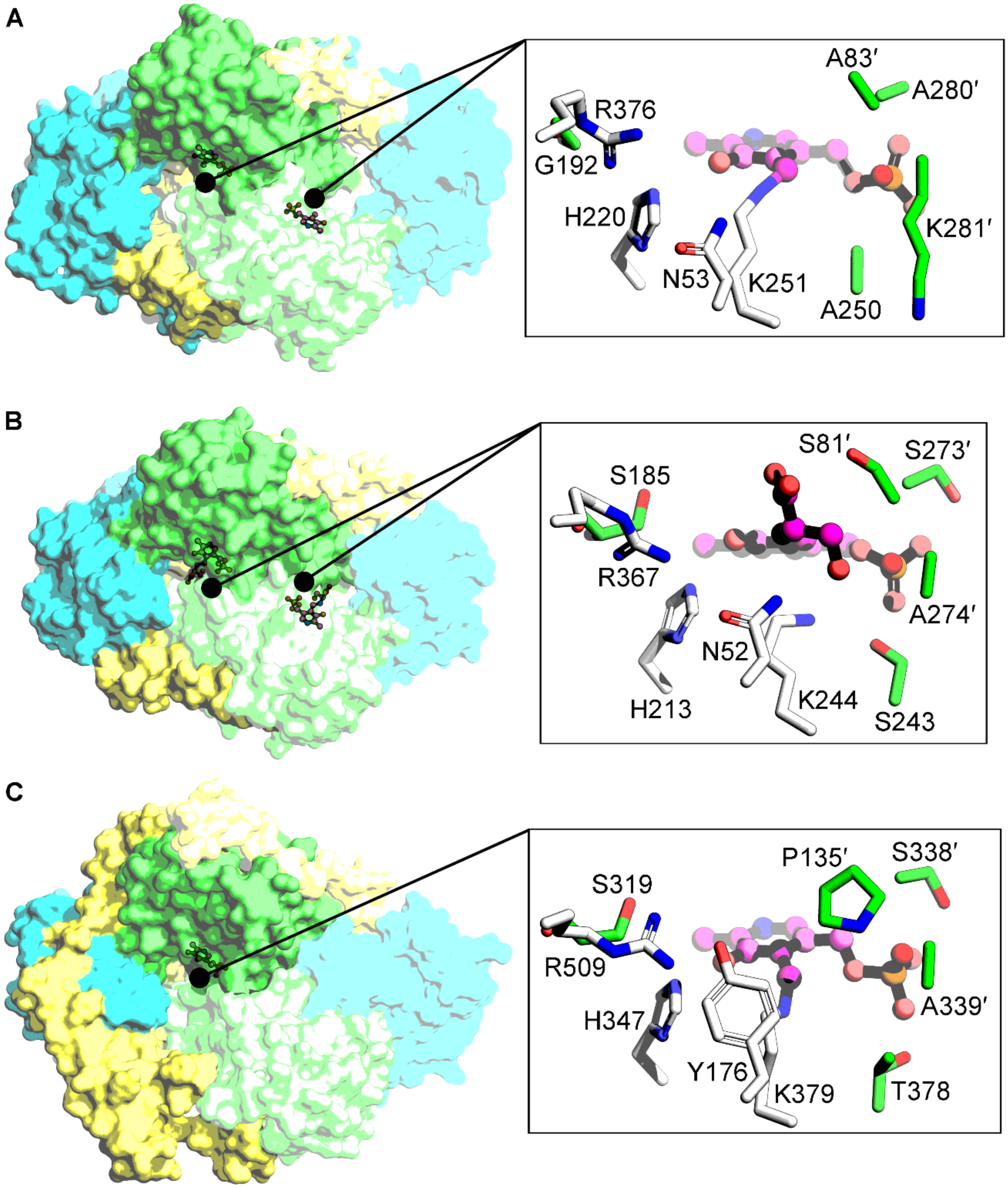

Figure S5. Active site comparison of the serine palmitoyltransferase (Spt) enzyme family. The external surface of *A*, *Alistipes finegoldii* SulA internal aldimine, *B*, *Sphingobacterium multivorum* Spt external aldimine (PDBID: 3A2B), and *C*, *Homo sapiens* Spt internal aldimine (PDBID: 7K0I). The SulA homodimer is structurally similar to the *S. multivorum* Spt homodimer and *H. sapiens* Spt heterodimer with R.M.S.D. values of 1.50 and 1.34 Å respectively. Electrostatic surface visualization is shown from -5 kT/e (red) to +5 kT/e (blue) of the solvent-accessible surface, calculated and displayed by APBS (63). The zoomed-in image shows residues at identical positions within the active sites of *A*, *B*, and *C*. Residues common to Spt enzymes are shown in white and other active site residues are shown in green. Residues marked with a prime symbol are from the opposite protomer.
